# Morphology and stable isotope analysis demonstrate different structuring of bat communities in rainforest and savannah habitats

**DOI:** 10.1098/rsos.180849

**Published:** 2018-12-19

**Authors:** Ara Monadjem, Adam Kane, Peter Taylor, Leigh R. Richards, Grant Hall, Stephan Woodborne

**Affiliations:** 1 Department of Biological Sciences, University of Eswatini, Private Bag 4, Kwaluseni, Eswatini; 2 Department of Zoology and Entomology, Mammal Research Institute, University of Pretoria, Private Bag 20, Hatfield 0028, Pretoria, South Africa; 3 School of Biology and Environmental Science and Earth Institute, University College Dublin, Dublin, Ireland; 4 School of Mathematical and Natural Sciences, University of Venda, Private Bag X5050, Thohoyandou 0950, South Africa; 5 School of Life Sciences, University of KwaZulu-Natal, Biological Sciences Building, South Ring Road, Westville Campus, Durban, KwaZulu-Natal 3630, South Africa; 6 Durban Natural Science Museum, PO Box 4085, Durban 4000, South Africa; 7 iThemba LABS, Private Bag 11, WITS 2050, Pretoria, South Africa

**Keywords:** bats, rainforest, savannah, morphometrics, community structure, stable isotope analysis

## Abstract

Bats play important ecological roles in tropical systems, yet how these communities are structured is still poorly understood. Our study explores the structure of African bat communities using morphological characters to define the morphospace occupied by these bats and stable isotope analysis to define their dietary niche breadth. We compared two communities, one in rainforest (Liberia) and one in savannah (South Africa), and asked whether the greater richness in the rainforest was due to more species ‘packing’ into the same morphospace and trophic space than bats from the savannah, or some other arrangement. In the rainforest, bats occupied a larger area in morphospace and species packing was higher than in the savannah; although this difference disappeared when comparing insectivorous bats only. There were also differences in morphospace occupied by different foraging groups (aerial, edge, clutter and fruitbat). Stable isotope analysis revealed that the range of *δ*^13^C values was almost double in rainforest than in savannah indicating a greater range of utilization of basal C_3_ and C_4_ resources in the former site, covering primary productivity from both these sources. The ranges in *δ*^15^N, however, were similar between the two habitats suggesting a similar number of trophic levels. Niche breadth, as defined by either standard ellipse area or convex hull, was greater for the bat community in rainforest than in savannah, with all four foraging groups having larger niche breadths in the former than the latter. The higher inter-species morphospace and niche breadth in forest bats suggest that species packing is not necessarily competitive. By employing morphometrics and stable isotope analysis, we have shown that the rainforest bat community packs more species in morphospace and uses a larger niche breadth than the one in savannah.

## 1. Introduction

Species richness is not evenly distributed across the landscape, but changes in relation to a variety of factors such as latitude, altitude and climate [1]. The distribution of species richness has been relatively well mapped at continental scales [2–5], and environmental correlates of species richness have been widely reported. For example, latitude, precipitation and topography are all important predictors of species richness of birds in South America [4], with greater species richness in the tropics than in zones further away from the equator [6]. How and why tropical communities contain a larger number of species than subtropical or temperate ones is still not resolved and numerous hypotheses have been advanced to address this question [6–8].

African bats show the typical pattern of increased species richness towards the equator [9], with higher species richness in rainforest than savannah habitats [10–13]. Why there should be greater species richness in rainforest habitats is not clear [14], although it has been suggested that forests have higher heterogeneity, hence allowing for greater niche partitioning [15]. Food, as a primary resource, is often the focus of such niche partitioning studies [16], and recent advances in stable isotope analyses have allowed novel insights into niche breadth and other dietary parameters that were previously difficult to study [15,17–20]. Since bats manipulate their prey or food item with their mouths, the structure of bat skulls may indicate the morphological space (morphospace) occupied by a particular community [21]. Various studies have shown that ecological (including trophic) relationships within animal communities can be based on relevant morphological characters [16,22,23]. Furthermore, these ecomorphological traits can be used to calculate indices of ‘species packing’ within a community, allowing for comparisons between communities [24]. In this respect, species packing refers to how different species occupy morphospace which may then be used to compare between communities.

Our primary objective was to test the relationship between species richness and community structure at two well-surveyed African mountains of equal size and height, one in the rainforest belt and the other in a savannah environment. The rainforest site was Mount Nimba that straddles Liberia, Guinea and Côte d'Ivoire and has been surveyed extensively with 59 species recorded to date [12]. The savannah site was the Soutpansberg Mountains in northern South Africa from where 45 species of bats have been recorded although some of these are restricted to lowland riparian habitats at Pafuri to the northeast of the mountain range [13,25–27]. Both mountains rise to just above 1700 m above sea level and cover a surface area of approximately 700 km^2^. However, Mount Nimba is situated approximately 7° north of the equator while the Soutpansberg Mountains lie 23° to its south. Both are located in regional zones of high bat species richness [9,11,28]. We specifically asked whether the greater richness at Mount Nimba was due to more species ‘packing’ into the same morphospace (based on craniodental characters) and trophic niche (based on stable isotopes) than bats from the Soutpansberg Mountains, or whether it was due to a larger morphospace and niche breadth at the former site. Since the packing in morphospace of tropical versus temperate bats does not appear to differ [14], we predict that morphospace metrics will be similar between Mount Nimba and Soutpansberg Mountains. With regard to niche breadth, we predict that when different bats exploit a similar available dietary niche (indicated by similar *δ*^13^C ranges) there would be fine-scale trophic partitioning of the realized niche (indicated by the range of *δ*^15^N values) to reduce competition. This prediction suggests that there should be a correlation between species packing and *δ*^15^N values. Conversely, greater species packing without greater dietary diversity implies a greater competition between species, and in this case we would anticipate that species packing would be controlled by ecosystem productivity. Hence, we anticipate that there will be a correlation between morphology and diet as a result of the evolutionary trajectory behind the species packing.

## 2. Material and methods

### 2.1. Study sites

**Figure 1.**
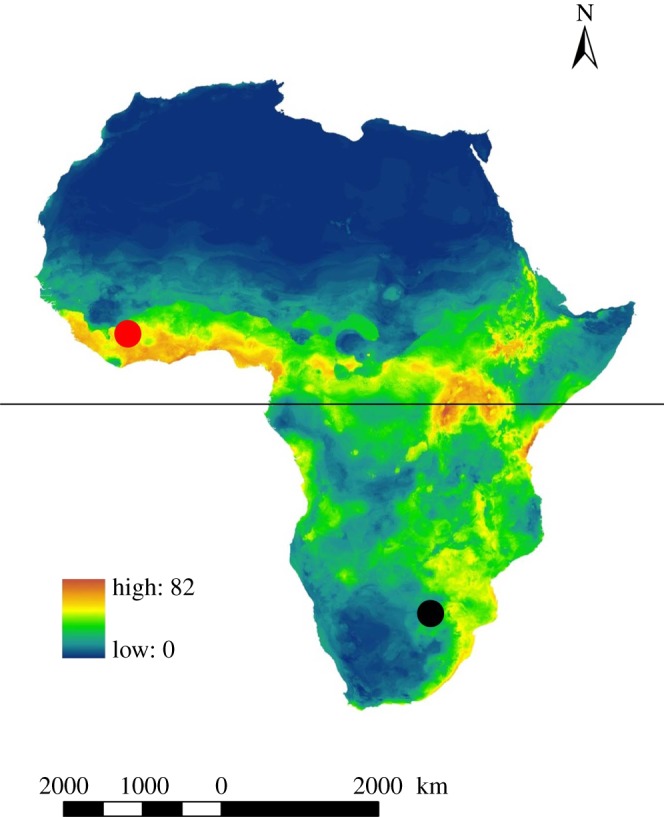
Map showing the location of Mount Nimba (Liberia) and Soutpansberg Mountains (South Africa) as red and black circles, respectively, overlaid on a bat species richness map for Africa modified from Monadjem *et al*. [29]. The black line represents the equator. Note that both sites are in zones of high species richness.

This study was conducted at Mount Nimba in Liberia and the Soutpansberg Mountains in South Africa (figure 1). Mount Nimba is situated in the transition zone between the tropical forest zone to the south and moist woodlands to the north and supports a rich biodiversity and high endemism ([12] and references therein). Highland areas above 1000 m are limited to a few isolated peaks and mountain ranges in West Africa, and as a result, levels of endemism are very high in these areas. Mount Nimba is approximately 40 km in length (straddling three countries), rising sharply from the plains below to 1768 m above sea level. There is an obvious rainfall gradient with the southern and southeastern slopes receiving more rain than the northern slopes. The rains fall mainly from April until November with a pronounced dry season from December to February [30]. Lowland evergreen forest covers the lower slopes of the mountain, giving way to montane forest above 1000 m, with a variety of natural forested habitats occurring in the transition zone; however, the high altitude zone above 1400 m is predominantly covered by grassland [31].

The Soutpansberg (and associated Blouberg and Makgabeng) Mountain Range situated in the Savanna Biome of northern South Africa comprises a recognized Centre for Plant Endemism [32] with some 3000 plant species (of which 44 are endemic) and 1066 genera [33,34]. Faunal diversity is also high. The Soutpansberg harbours 33% of South Africa's reptiles, 60% of its mammals, 75% of its birds, 50% of the world's spider families and exceptionally diverse ant communities [35–37]. Variation in aspect and topography within the Soutpansberg results in strong latitudinal, longitudinal and elevational gradients in climate and vegetation. Owing to a rain shadow on the northern slopes, precipitation varies from 367 mm on the northern slopes to greater than 3000 mm (of which one third falls as mist) on the southeastern slopes [33]. Most of the Soutpansberg is covered by various types of savannah including thickets (Soutpansberg Mountain Bushveld), with small patches of temperate grasslands occurring at higher elevations (Soutpansberg Sourveld) and small patches of Mistbelt forest occurring at the bases of cliffs on the southern slopes (Soutpansberg Mistbelt Forest) [38].

### 2.2. Data collection

The sampling strategy was designed around the collection of voucher specimens for museum collections, and the decision to compare morphometric and isotope analysis was *post hoc*. Two intensive one-month-long bat surveys were conducted at Mount Nimba in December 2010 to January 2011, and in December 2011 to January 2012. A further two-week survey was conducted in March 2013. Daily searches were made in an area of approximately 100 km^2^ for bat roosting sites, including adits, culverts (under roads and the railway), tunnels, buildings, hollow trees, natural caves and cavities. When present, bats were trapped by blocking the entrance to the roost with a mist net. Each night a new site was visited where up to five mist nets (12 × 2.5 m, with 16 mm mesh size, Ecotone, Poland) were erected in suitable locations to maximize capture success. A two-bank harp trap (3′ Cave Catcher from Bat Conservation and Management; www.batmanagement.com) was set up alongside the mist nets at all sites. Nets and the harp trap were in place at least 30 min before sunset and were generally removed at 22.00 or once bat activity had died down, based on reduced capture rates.

As elaborated in more detail in Taylor *et al*. [25], specimens of bats from the Soutpansberg were obtained between January 2010 and April 2013 from 30 localities (covering approx. 300 km^2^) along the southern aspect of the Blouberg and Soutpansberg Mountains between altitudes of 600 and 1747 m using a two-bank harp trap (Faunatech, Australia) set for 29 nights and 54 mistnet-nights (mistnets of 9 and 6 m lengths supplied by Ecotone, Poland), as well as *ad hoc* searches for day and night roosts. We sampled for approximately 2 h after sunset or until there was no further bat activity. Harp traps were deployed from sunset till sunrise along presumed flyways.

All captured bats were sexed and age (adult versus juvenile) was determined by the degree of ossification of the wing bones (fused in adults). Mass (g) was recorded using a Pesola balance while forearm length (mm) was measured using either vernier or digital callipers. In order to reliably identify each bat, voucher specimens (alcohol skins and skulls) were obtained for each species collected in addition to soft tissues (for possible DNA sequencing) and these were deposited in the mammal collection of the Durban Natural Science Museum. For most species, this involved taking a single male and a single female specimen. For certain groups (particularly the pipistrelloid genera of *Neoromicia*, *Hypsugo* and *Pipistrellus*) additional voucher specimens were collected. This was necessitated by the fact that these species are virtually impossible to identify correctly in the field and in fact several new species have been described to science from the Mount Nimba region based on our collections [12,39,40]. Identification was based on Monadjem *et al*. [13].

Appropriate permits for fieldwork were received for the sites in Liberia and South Africa. Animal handling was conducted in accordance with the guidelines from the American Society of Mammalogists [41]. See Ethics statement for details.

### 2.3. Morphometric analysis

We took five external and six craniodental measurements of all specimens in order to compare community structure of bats at Mount Nimba and Soutpansberg Mountains in morphospace [14,21,42]. The following standard external measurements were taken in the field: total body length, tail length, forearm length and hindfoot length. Forearm length was taken with callipers to the closest 0.1 mm; all other measurements were at an accuracy of 1 mm. In addition, body mass was taken with a Pesola spring balance to the closest 1 g. Four cranial and two dental measurements were taken with callipers to the closest 0.01 mm following Monadjem *et al*. [39]: greatest skull length (GSKL), from the posterior-most point of the cranium to the anterior-most point of the incisors; greatest zygomatic breadth (ZYGO), the greatest width across the zygomatic arches; greatest braincase width (GBW), lateral braincase width taken posterior to the posterior insertion of the zygomatic arches; greatest mandible length (MAND), taken from the posterior-most point of the condylar processes to the anterior-most point of the incisors; complete upper canine-molar tooth row (C-M3), taken from the anterior-most point of the alveolus of the canine to the posterior-most point of the third molar; and width across upper canines (C–C), taken across the outer-most points of the alveoli of the canines.

### 2.4. Stable isotope analysis

Voucher specimens were collected as part of another study [12], from which a small wing-punch material was preserved in 99% ethanol. All isotope samples referred to in this study are based on specimens deposited in the Durban Natural Science Museum. Voucher specimens are made available for further research by the museum, and in this case the need for a very small aliquot for the isotope analysis did not compromise the collection which remains intact.

Samples were dried at 70°C to remove the ethanol. Ethanol has little effect on *δ*^15^N values, but it has the potential to affect *δ*^13^C values [43]. The wing-punch tissue retains insignificant amounts of fat and so lipid extraction was not required. Sub-aliquots of approximately 0.6 mg were weighed into tin cups that had been pre-cleaned with toluene. The *δ*^15^N and *δ*^13^C values were measured on the same sample aliquots using a Delta V Plus stable light isotope mass spectrometer coupled to a Thermo Series 1112 flash elemental analyser by a ConFloIV interface (all equipment supplied by ThermoFinnigan, Germany). Two laboratory standards were used to correct the isotopic values: dl-Valine (*δ*^15^N = −6.15‰ and *δ*^13^C = −10.57‰) and Merck Gel (*δ*^15^N = 7.89‰ and *δ*^13^C = −20.26‰). Standards and blank samples were run after every 10 unknown samples. Precision on replicates was less than 0.1‰ for both isotopes and for both standards. The %N and %C for the samples were also calculated from the MS peak areas, and the resulting C/N ratio (4.0 ± 0.2 across all unknowns) indicates that there was insignificant influence of lipids in the analysis [44].

### 2.5. Data analysis

We performed a principal component analysis (PCA) on each of the bat communities using the morphological measures described above. In order to control for the size of the bats, we first regressed each of the 10 measurement variables (external and craniodental) against body mass and calculated the residuals which were then used in the PCA analysis. We used the first 10 principal components of our morphological analysis to extract morphospace coordinates. This was done both with and without the fruitbats (family Pteropodidae). We used these coordinates to calculate the nearest neighbour distances and distance to centroids in morphospace as two measures of species packing. We first compared the two communities directly. Then, we accounted for unequal species richness between the communities by bootstrapping and calculating the distance to centroid 500 times. Each subsample included 22 species to control for effects of species richness. A Wilcoxon rank sum test was used to test for significant differences in the nearest neighbour distance and distance to centroids for bats at Mount Nimba and Soutpansberg Mountains [45]. For the bootstrapped sample, we took into account multiple testing, which could lead to Type 1 error, by looking at the distribution of *p*-values rather than a single value. Bat species were assigned to four functional foraging groups: open air foragers (open), clutter-edge (edge), clutter and fruitbats [46,47]. We then constructed convex hulls around the morphospace mapped out by the 10 PCA axes for each of the communities and calculated the volume of the resulting multi-dimensional shape as a measure of the extent of morphospace covered by the bats in each community. These analyses were performed in R v. 3.4.4 (www.r-project.org); with the package *spatstat* used for calculating the distance to nearest neighbour, *dispRity* for distance to centroid and *sp* for convex hull area calculation [45,48,49]. All the relevant R code is made available in the electronic supplementary material.

With respect to stable isotope analyses, we have made the assumption that the diet to tissue discrimination factor is relatively similar in all bat species, and that the relative dietary niches are reasonably assessed by direct comparison of the isotopic values. While *δ*^15^N values are expected to vary in response to the trophic enrichment, and *δ*^13^C in response to the range in C_3_ and C_4_ assimilation at the base of the food web [50], we acknowledge that there are many different dietary combinations that may yield a generalist isotopic combination. By contrast, species whose isotope values plot near the limits of the isotopic space are almost certainly exploiting a narrow range of dietary items. The argument of equi-finality of a generalist diet versus a specific dietary source that yields a median isotope value in the bats is addressed by considering the intra-species isotope variability. A specialist diet at species level may have a median isotope value, but all individuals will covary within a narrow range. However, we do not have sufficient numbers of replicates of each species to test this, but we assume that a generalist diet at species level will result in greater diversity of isotope values at the individual level. We calculated the six Layman metrics that summarize community structure based on: (1) range of *δ*^15^N (NR) representing the number of trophic levels; (2) range of *δ*^13^C (CR) estimating the diversity of basal resources; (3) mean distance to centroid (CD) indicating the average trophic diversity of the community; (4) mean nearest neighbour distance (MNND) indicating the level of species clustering and packing; (5) standard deviation of the nearest neighbour distance (SDNND) representing the evenness of species packing; and (6) total area of the convex hull encompassing the data points (TA) providing an indication of niche breadth [51]. In addition, we also calculated two metrics following Jackson *et al.* [52]: (7) area of standard ellipse (SEA); and (8) corrected area of standard ellipse (SEA_c_) for small sample sizes. These last two metrics provide further information on niche breadth and are thought to be better descriptors than the convex hull method [52]. All stable isotope analyses were conducted in the R packages *siar* and *SIBER* [52].

## 3. Results

**Figure 2.**
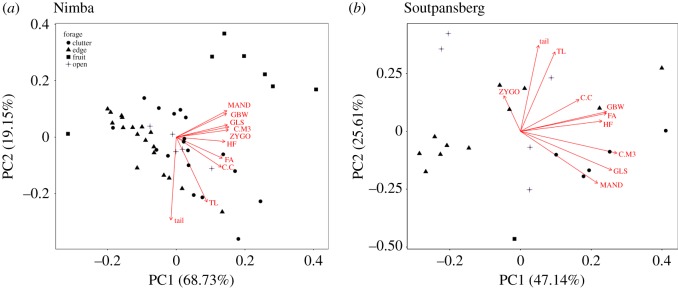
Principal components analysis (PCA) plots of bat communities by the four functional groups of open, edge, clutter and fruitbat as defined in this study: (*a*) the rainforest (Nimba) community; and (*b*) the savannah (Soutpansberg) community. Note the distinct area of morphospace occupied by fruit bats in the forest site.

A total of 48 and 23 species of bats were included in the morphometric analysis from Mount Nimba and Soutpansberg Mountains, respectively (electronic supplementary material, table S1). The mean distance to the nearest neighbour was significantly shorter for the rainforest bat community (Nimba) than for the savannah community (Soutpansberg), indicating higher species packing in morphospace at the rainforest site (table 1). Further, the rarefied sample did not change the significance of our results. However, this relationship was not significant when fruitbats, which occupy a different portion of morphospace from insectivorous bats (figure 2*a*), were removed from the analysis. This demonstrates that species packing was similar for insectivorous bats (table 1). Distance to centroid did not differ significantly between the two bat communities, with or without fruitbats (table 1). The morphospace area was 50% larger for the rainforest bat community compared with the savannah community, but they were similar (only 9% larger for the rainforest community compared with the savannah community) when fruitbats were removed from the analysis (table 1). Hence, it would appear that fruitbats are driving the differences in morphospace and species packing between rainforest and savannah bat communities.

**Table 1.** The mean (±s.d.) distance to nearest neighbour, median distance to centroid at Nimba (rainforest) and Soutpansberg (savannah), based on PCA conducted on residuals of 10 craniodental and external measurements regressed against body mass. Observed and rarefied bootstrapped medians are shown. The values for distance to centroid are based on bootstrapped rarefied samples to account for unequal species richness. Also shown are the minimum convex polygon areas for bat communities which are calculated on the first two PCA axes. The analyses were conducted on either the entire community or just the insectivorous bats (i.e. excluding fruitbats). n.s., non-significant at *α* = 0.05.

community	metric	statistics for Nimba	statistics for savannah	Wilcoxon rank test statistic (*p*-value)
all bats	distance to nearest neighbour	1.19 (±0.681)	1.87 (±1.05)	*W* = 278 (<0.002)
all bats	distance to centroid (observed and bootstrapped)	21.09 (19.47)	18.25 (17.55)	n.s.
all bats	minimum convex polygon area	46.68	31.24	—
insectivorous bats	distance to nearest neighbour	1.51 (±0.598)	1.78 (±0.831)	n.s.
insectivorous bats	distance to centroid (observed and bootstrapped)	17.45 (17.20)	18.45 (17.54)	n.s.
insectivorous bats	minimum convex polygon area	33.07	30.47	—

**Figure 3.**
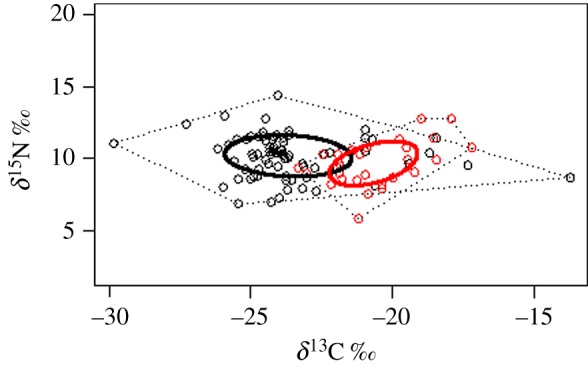
Mean *δ*^15^N and *δ*^13^C values of all bats recorded at Mount Nimba (forest—black circles) and Soutpansberg Mountains (savannah—red circles). The dotted lines represent the convex hulls and the solid lines the standard ellipse areas for the bat communities at the two sites. Note the larger size of the standard ellipse area in forest compared with savannah.

A total of 39 and 17 species of bats were included in the isotope analysis from Mount Nimba and Soutpansberg Mountains, respectively (electronic supplementary material, table S1). The range in *δ*^15^N values was similar at the two sites, extending from 6.9‰ to 14.3‰ (range = 7.4‰) and 5.9‰ to 12.8‰ (range = 6.9‰) for Nimba and Soutpansberg, respectively, and encapsulating at least two (perhaps three) trophic levels, assuming that increase in *δ*^15^N values of 3.4‰ is the equivalent of a trophic level ([53]). By contrast, the range in *δ*^13^C values was different between the two sites, extending from −29.8‰ to −13.7‰ (range = 16.1‰) and −23.3‰ to −17.2‰ (range = 6.1‰) for Nimba and Soutpansberg, respectively (figure 3). The convex hull area covered by the Nimba bat community (62.8‰^2^) was triple that of the Soutpansberg bat community (21.1‰^2^), indicating a broader niche complex. The distance to centroid was similar at Mount Nimba (2.04‰) and Soutpansberg (1.90‰); however, the MNND was slightly smaller at the former (0.50‰) compared with the latter (0.61‰), suggesting tighter species packing at Mount Nimba.

**Figure 4.**
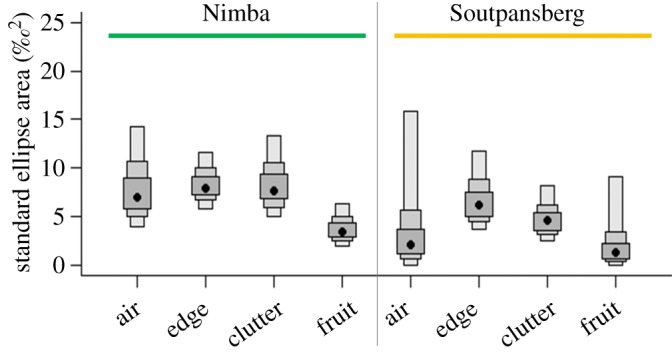
Density plots showing the credibility intervals of the standard ellipse areas (SEA) for bats based on stable isotope analysis. Black circles represent the modal SEA, and boxes indicate the 50%, 75% and 95% credible intervals. ‘Nimba’ refers to the forest habitat versus ‘Soutpansberg’ which refers to the savannah site. Note the larger standard ellipses in the forest compared with the savannah for each one of the bat foraging groups.

**Figure 5.**
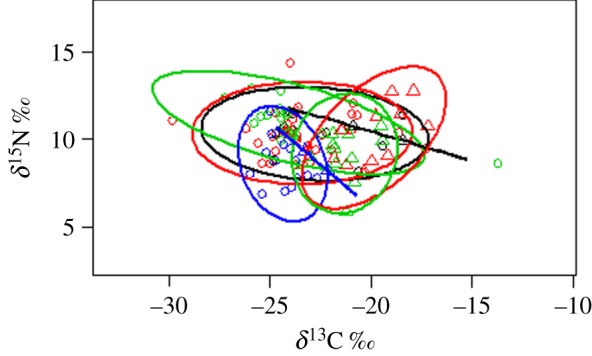
Mean *δ*^15^N and *δ*^13^C values of the different bat foraging groups recorded at Mount Nimba (forest) and Soutpansberg Mountains (savannah). The outlines represent standard ellipse areas for each foraging group at each site with the black ellipse referring to aerial foragers, red to edge foragers, green to clutter foragers and blue to fruit bats. Note that there are two ellipses of each colour, one representing the forest site (associated with circles) and one the savannah site (associated with triangles), with the former always on the left side of the latter. The blue and black straight lines are in fact also ellipses but at this scale appear as straight lines due to the small sample size of these two foraging groups in savannah (see electronic supplementary material, table S2). For each pair of ellipses of the same colour (e.g. foraging group), note that the range in *δ*^15^N is similar, but the range in *δ*^13^C is larger in the forest site.

Dietary specialization is revealed in the intra-group isotope values. Within each foraging group, the standard ellipses were larger at Mount Nimba compared with Soutpansberg (figure 4). In general, foraging groups at Nimba had larger ranges in *δ*^15^N and *δ*^13^C values, covered larger areas of isotopic space, had smaller mean distances to centroid, and smaller mean nearest neighbouring distances than functional groups at Soutpansberg (figure 5, electronic supplementary material, table S2). The exception was the clutter foraging group that had similar *δ*^15^N ranges at the two sites, but larger mean distances to centroid and mean nearest neighbouring distances at Soutpansberg than at Nimba (electronic supplementary material, table S2). Typically, for the same bat foraging group, the standard ellipses at Nimba covered lower values of *δ*^13^C (i.e. situated on the left side of the graph) than those at Soutpansberg (figure 5). By contrast, based on the range in *δ*^15^N, each foraging group appeared to cover the same number of trophic levels at Nimba and Soutpansberg (figure 5). Fruit bats occupied lower trophic levels (lower values of *δ*^15^N), but there was no obvious difference in the trophic levels of the remaining foraging groups.

## 4. Discussion

Mount Nimba in the equatorial rainforest belt harboured a greater *α* diversity of bats than the Soutpansberg Mountains situated in tropical savannah. The higher species richness of vertebrate taxa in the African rainforest belt compared with savannahs is well known [5,9], but how these extra species pack into the rainforest zone is not well understood [14]. In this study, we have demonstrated that the morphospace of the bats in rainforest was larger than in savannah, although this difference disappeared when only insectivorous bats were compared (i.e. fruitbats were excluded). In addition, the packing of species in morphospace (based on nearest neighbour distances) was greater for bats in forest than in savannah (but only when fruitbats were included in the analysis), even though the distance to centroid did not differ between the two sites. Isotopic values of the two sites also differed, with the forest bat community having a wider niche breadth than the savannah community. Hence, the greater species richness in the rainforest site is achieved morphologically by the presence of fruitbats adding to morphospace of the bat community; and by a greater niche breadth. The apparent discrepancy between the nearest neighbour distances and the distance to centroid metrics (for the entire community including fruitbats) can be explained by the greater clustering in morphospace of the rainforest bat community compared with the one in the savannah. As a result, there is a large amount of unoccupied morphospace in the rainforest bat community which is apparent in a visual comparison of figure 2*a*,*b*. We do not know why this should be, but it is worth noting that the ‘empty’ space lies between fruitbats on the one hand and the remaining insectivorous bats on the other. Therefore, we partially supported our first prediction (that morphospace and species packing should be similar in rainforest and savannah), corroborating a previous study on species packing of bats [14]. However, these metrics were only similar between rainforest and savannah for insectivorous bats.

The two bat communities studied here, although both African, shared very few species in common. Of the 71 species that we included in our analyses, just 4 (6%) species were shared by both sites (*Chaerephon pumilus*, *Mops condylurus*, *Neoromicia nana* and *Rhinolophus simulator*). This is not surprising, because tropical African rainforests have a distinct bat fauna [10]. However, of the 27 genera, 9 (33%) were shared, with 47% of the insectivorous genera occurring at both sites (i.e. if fruitbats are excluded). Despite these differences in the composition of the rainforest and savannah communities, morphospace and species packing metrics were similar for insectivorous bats. This suggests that there are basic underlying constraints that structure the insectivorous component of African bat communities.

The four foraging groups in this study occupied different regions of morphospace, with that of fruitbats not overlapping that of microbats. The edge foragers also separated with little overlap. Surprisingly, the clutter and aerial foragers overlapped considerably in morphospace. Clutter foragers typically have short, rounded wings with low wing-loading and low aspect ratio while aerial foragers have long wings with high wing-loading and high aspect ratio [54–56]. However, we defined morphospace on a variety of features that included the size and shape of the cranium (as an indication of the range of prey available to the bat). Edge foragers were typically smaller than clutter or aerial foragers and had relatively longer tails, hence the separation in morphospace.

Isotopic niches also differed between the foraging groups. In the rainforest site (Nimba), the non-fruitbat foraging groups had similar niche breadth as defined by standard ellipse areas that were double that of fruitbats. The narrower niche breadth (as defined by stable isotopes) of fruitbats is not surprising because isotopic signatures of fruits are likely to be more similar than those of arthropods, that may comprise distinct trophic levels, which form the diets of the other three foraging groups [57]. By contrast, the foraging groups in the savannah site (Soutpansberg) showed greater variability in niche breadth, perhaps as a result of a skew in species richness between the groups. The edge foragers in savannah had the largest niche breadths, with a mean value approaching that of edge species from rainforest, followed by clutter foragers that had niche breadth of just over half of those from Nimba. Edge species by definition forage in the zone between cluttered and open habitats [47], and therefore the similarity between the two communities should not be surprising. By comparison, a forest provides greater structural complexity than a savannah allowing a greater number of clutter foragers to coexist. This is seen by the greater diversity of clutter foragers in rainforest which includes six genera (*Doryrhina*, *Hipposideros*, *Macronycteris*, *Nycteris*, *Kerivoula* and *Rhinolophus*) compared with just two genera in savannah (*Hipposideros* and *Rhinolophus*). Aerial foragers and fruitbats had the lowest niche breadths, almost certainly as a result of the inclusion of just two and one species in these two groups, respectively. We recorded seven species of fruitbat (see electronic supplementary material, table S1) at the rainforest site compared with just one species (the widespread *Epomophorus wahlbergi*) at the savannah site. Presumably species richness and niche breadth in fruitbats are causally related, with the rainforest site providing a greater abundance and diversity of fruiting trees than the savannah site. In summary, niche breadth was greater in the rainforest bat community compared with the savannah community, corroborating our second prediction. To the best of our knowledge, this is the first direct comparison of niche breadth in forest and savannah bat communities.

The isotope values of the bat community in the forest habitat were depleted in ^13^C compared with that in the savannah, suggesting a greater proportion of basal resources were obtained from C_3_ resources by the forest community. However, this is not to say that forest bats were only eating forest insects and savannah bats only grassland species because the *δ*^13^C range of the forest community indicated both forest and grassland resource use. A study investigating stable isotope values in terrestrial small mammals in nearby forest and grassland sites in South Africa reported greater enriched *δ*^13^C values in the latter habitat [18]. In our study, the forest community, although spanning the entire *δ*^13^C range of the savannah community, nevertheless has a far lower (more negative) mean *δ*^13^C value, indicating diets that include a greater proportion of forest products. This greater range in *δ*^13^C at the rainforest site may be associated with clear-cutting of forest in this region, driven by mining, timber interests and slash-and-burn agriculture which has transformed forest habitat into more open ‘savannah’ habitats [12]. In any case, Mount Nimba is situated in the transition zone between rainforest and savannah [10], where these two interdigitating biomes come into close proximity of each other. Assuming that trophic enrichment for bats is similar to other mammals (around 3–4‰ [44]), then both bat communities appear to support at least two distinct trophic levels, as has been reported for bat communities in forests of Madagascar [15,58]. The range of diets of African bats varies from frugivorous in the pteropodids [13], to the consumption of a wide range of arthropod orders in the molossids, nycterids and vespertilionids [59,60], and a single carnivorous species *Nycteris grandis* [61]. The isotopic values we present in this study reconcile with the inferred diets of the few African bats that have been studied to date.

Our analysis aims to distinguish the forcing that underlies species packing through the physical adaptation (morphospace) and dietary preferences (isotope space) between the high *α* diversity of bats at Mount Nimba and the Soutpansberg Mountains. When fruitbats are excluded from the analysis, then the higher species richness of microbats in the rainforest (Mount Nimba) may be attributable to dietary partitioning as the niche breadth (defined by isotope analysis) is larger here than in the savannah (Soutpansberg Mountains). We conclude that species packing is not the result of an evolutionary process to reduce inter-species competition, but rather the result of higher productivity at Mount Nimba than at the Soutpansberg Mountains. If the evolution of diet and morphology in sympatric bat communities is driven by intensification of resource use, then the complete separation of fruitbats without intermediate forms between them and insectivorous bats that are evident in this study remains enigmatic.

## 5. Conclusion

We show that a tropical forest bat community occupied a larger morphospace area and had a greater niche breadth as measured by stable isotope analysis than a comparable community in the savannah zone. Hence, our study has demonstrated the power of using morphological studies in associating with stable isotope analysis in elucidating the structure of tropical bat communities. To the best of our knowledge, this is the first study to compare the bat communities of tropical rainforest and savannah habitats in terms of morphospace and isotopic niche breadth.

## Supplementary Material

R code

## Supplementary Material

Supplementary Tables

## Supplementary Material

Foraging groups_Nimba_Soutpans.csv

## Supplementary Material

nimba.csv

## Supplementary Material

residBatData.csv

## Supplementary Material

sout.csv

## Data accessibility

All the various R code is made available as electronic supplementary material.
