# Supplementary material for: Morphology and stable isotope analysis demonstrate different structuring of bat communities in rainforest and savannah habitats
Source: R Soc Open Sci. 2018 Dec 19;5(12):180849. doi: 10.1098/rsos.180849 (PMC6304110; doi:10.1098/rsos.180849)
Supplement: R code [file rsos180849supp1.pdf]

morphospaceAnalysis.R

Adam

Fri Nov 30 16:01:54 2018

```
# Morphology and stable isotope analysis demonstrate different structuring of bat communities in
# rainforest and savannah habitats

# Ara Monadjem, Adam Kane, Grant Hall, Peter Taylor, Leigh Richards, Stephan Woodborne

# data files
# 1. sout.csv morphological data for Soutpansberg bats
# 2. nimba.csv morphological data for Nimba bats
# 3. Foraging groups_Nimba_Soutpans.csv assigns the foraging groups
# 4. residBatData.csv residuals of trait values against body mass

rm(list=ls())
warnings=F
```

```
# load in bat data
```

```
sout<-read.csv("sout.csv",header = T, sep = ",") # Soutpansberg
nimba<-read.csv("nimba.csv",header = T, sep = ",") # Nimba
batSpecies <- read.csv("Foraging groups_Nimba_Soutpans.csv")
sout$forage <- cbind(as.character(batSpecies$Foraging.group[batSpecies$Location=="Soutpansberg"]))
nimba$forage <- cbind(as.character(batSpecies$Foraging.group[batSpecies$Location=="Nimba"]))

head(nimba)
```

```
##   Mass   FA    TL Tail   HF   GLS  ZYGO   GBW  C.M3  C.C  MAND
## 1 12.8 42.0 109.0 35.5  8.5 18.85 11.31  9.39  6.98 4.84 12.84
## 2 74.0 83.5 155.0  0.0 22.0 44.29 23.57 17.14 15.21 8.12 34.44
## 3  8.0 43.7 106.0 50.0  8.0 16.18  9.02  7.80  6.07 4.72 11.95
## 4  7.7 39.7  97.7 44.7  6.3 12.18  8.64  7.51  4.25 4.23  9.02
## 5  5.8 43.5  74.0 27.0  6.5 17.08  9.51  7.78  6.18 3.89 10.92
## 6 39.5 67.8 120.0 44.0 16.0 28.88 15.40 11.48 10.26 7.18 19.22
##                 Row.Labels fruit  forage
## 1       Chaerephon_pumilus     0    Open
## 2     Epomops_buettikoferi     1   Fruit
## 3 Glauconycteris_argentata     0    Edge
## 4   Glauconycteris_poensis     0    Edge
## 5      Hipposideros_beatus     0 Clutter
## 6     Hipposideros_cyclops     0 Clutter
```

```
head(sout)
```

```
##   Mass   FA    TL Tail   HF   GLS  ZYGO   GBW  C.M3  C.C  MAND
## 1 12.0 39.0  86.7 31.5  8.0 18.30  9.80  9.07  6.62 5.59 12.55
## 2 78.5 77.5 139.0  2.0 23.2 46.46 25.90 16.73 16.29 8.96 36.86
## 3 19.8 47.5 116.0 49.1 10.0 19.27 12.76  9.27  6.71 5.94 14.33
## 4  6.4 47.3  86.5 30.0  9.0 17.51  8.81  8.16  5.96 3.86 11.00
## 5  6.6 37.2 103.0 42.0  9.0 15.50  8.50  7.51  5.00 4.31 10.38
## 6  8.9 45.2 110.7 51.3 10.0 15.24  8.12  7.73  5.75 4.32 11.19
##               Row.Labels fruit  forage
```

```
## 1     Chaerephon_pumilus     0    Open
## 2  Epomophorus_wahlbergi     1   Fruit
## 3  Eptesicus_hottentotus     0    Edge
## 4    Hipposideros_caffer     0 Clutter
## 5    Laephotis_botswanae     0    Edge
## 6 Miniopterus_natalensis     0    Edge
```

```
# REMOVE FRUIT BATS FROM HERE IF NEEDED
```

```
#nimba<-nimba[!(nimba$fruit==1),]
#sout<-sout[!(sout$fruit==1),]
```

```
# get rid of the column for fruit
```

```
nimba <- subset(nimba, select = -c(fruit))
sout <- subset(sout, select = -c(fruit))
```

```
# get residuals of trait ~ Mass to remove the effect of mass
# first for the Soutpansberg bats
```

```
soutLMResid <- lapply( sout[,-(c(12:13))], function(x)
  resid(summary(lm(x~sout$Mass)) ))
```

```
## Warning in summary.lm(lm(x ~ sout$Mass)): essentially perfect fit: summary
## may be unreliable
```

```
# add the residuals for each of the measures to the dataframe
sout$FA <- soutLMResid$FA
sout$TL <- soutLMResid$TL
sout$Tail <- soutLMResid$Tail
sout$HF <- soutLMResid$HF
sout$GLS <- soutLMResid$GLS
sout$ZYGO <- soutLMResid$ZYGO
sout$GBW <- soutLMResid$GBW
sout$C.M3 <- soutLMResid$C.M3
sout$C.C <- soutLMResid$C.C
sout$MAND <- soutLMResid$MAND
head(sout)
```

```
##   Mass         FA         TL       Tail         HF        GLS        ZYGO
## 1 12.0 -3.9175610 -10.469611  -4.469339 -1.1854227  0.5406224  -1.3025166
## 2 78.5 -7.4577093 -23.620975 -22.386064  1.3440258  2.1468374 -10.3578276
## 3 19.8 -0.3485859  11.153387  14.489300 -0.6715927 -1.6039569  -1.2930644
## 4  6.4  7.9226620  -5.157917  -6.944773  0.8815711  1.9867306  -0.1741747
## 5  6.6 -2.3037745  11.145237   5.090064  0.8434642 -0.1031304  -0.5598297
## 6  8.9  4.2422053  16.581506  14.790688  1.4052346 -1.2815320  -1.8098631
##           GBW       C.M3        C.C        MAND             Row.Labels
## 1  0.71399827  0.2158539  0.6536821  0.03940742     Chaerephon_pumilus
## 2 -0.86087844  0.1296216 -0.8099869  3.09304991  Epomophorus_wahlbergi
## 3 -0.16919028 -0.8384861  0.4367254 -0.67381948  Eptesicus_hottentotus
## 4  0.58167209  0.3774314 -0.6692721  0.27941647    Hipposideros_caffer
## 5 -0.09610197 -0.6119107 -0.2338095 -0.40451242    Laephotis_botswanae
## 6 -0.19550372 -0.1993443 -0.3909890 -0.32969471 Miniopterus_natalensis
##    forage
## 1    Open
## 2   Fruit
## 3    Edge
```

```
## 4 Clutter
## 5    Edge
## 6    Edge
```

```r
# now for the Nimba bats
nimbaLMResid <- lapply( nimba[,-(c(12:13))], function(x)
  resid(summary(lm(x~nimba$Mass)) ))
```

```
## Warning in summary.lm(lm(x ~ nimba$Mass)): essentially perfect fit: summary
## may be unreliable
```

```r
# add the residuals for each of the measures to the dataframe
nimba$FA <- nimbaLMResid$FA
nimba$TL <- nimbaLMResid$TL
nimba$Tail <- nimbaLMResid$Tail
nimba$HF <- nimbaLMResid$HF
nimba$GLS <- nimbaLMResid$GLS
nimba$ZYGO <- nimbaLMResid$ZYGO
nimba$GBW <- nimbaLMResid$GBW
nimba$C.M3 <- nimbaLMResid$C.M3
nimba$C.C <- nimbaLMResid$C.C
nimba$MAND <- nimbaLMResid$MAND
head(nimba)
```

```
##   Mass        FA         TL       Tail         HF        GLS       ZYGO
## 1 12.8 -1.317807  13.606494   1.211198 -0.3075211  0.2056403  0.3605091
## 2 74.0 16.830329  25.734734 -25.835570  7.3134089 13.4217885  6.8967254
## 3  8.0  2.213712  13.263102  15.048199 -0.3464175 -1.5056262 -1.4805667
## 4  7.7 -1.671818   5.129140   9.706762 -2.0175986 -5.4457053 -1.8325090
## 5  5.8  2.853158 -17.519285  -8.255675 -1.6350784 -0.1662067 -0.7848098
## 6 39.5 14.294370   9.829108  13.399127  4.6275906  4.9026854  1.9533681
##          GBW        C.M3          C.C       MAND              Row.Labels
## 1  0.7248041  0.32006067 -0.002110207 -0.4525412       Chaerephon_pumilus
## 2  5.0844676  4.53465868  1.055898679 11.0128526     Epomops_buettikoferi
## 3 -0.5992871 -0.27500584  0.052163606 -0.5476702 Glauconycteris_argentata
## 4 -0.8726678 -2.07532250 -0.426944280 -3.4279907   Glauconycteris_poensis
## 5 -0.4974123 -0.02066132 -0.697960896 -1.2133543      Hipposideros_beatus
## 6  1.3356867  1.84824314  1.368491709  1.5059884     Hipposideros_cyclops
##    forage
## 1    Open
## 2   Fruit
## 3    Edge
## 4    Edge
## 5 Clutter
## 6 Clutter
```

```r
# PCA analysis on Soutpansberg bats
```

```r
# prcomp - performs a principal components analysis on
# the given data matrix and returns the results as an object
# of class prcomp.
# With parameter scale. = T, we normalize the variables to
# have standard deviation equals to 1.
# the [,-12] argument removes the column with the species names
pcaSout<-prcomp(sout[,-c(1,12,13)],scale. = T)
# pcaSout<-prcomp(soutt[,-12])
```

```r
# plot the PCA
# The parameter scale = 0 ensures that arrows are scaled to represent the loadings.
# biplot(pcaSout, scale = 0)
# can also plot with the species names
row.names(sout) <- sout$Row.Labels
# biplot(pcaSout<-prcomp(sout[,-c(1,12,13)],scale. = T))

#compute standard deviation of each principal component
std_dev <- pcaSout$sdev
#compute variance
pr_var <- std_dev^2
#proportion of variance explained
prop_varex <- pr_var/sum(pr_var)
# remove exponential notation
options(scipen = 999)
# show proportion of variance explained
prop_varex
```

```
##  [1] 0.471417292 0.256055709 0.123193173 0.078781366 0.035257862
##  [6] 0.015900547 0.011328846 0.003570385 0.002622523 0.001872296
```

```r
# alternatively just run summary
summary(pcaSout)
```

```
## Importance of components:
##                           PC1    PC2    PC3     PC4     PC5    PC6     PC7
## Standard deviation     2.1712 1.6002 1.1099 0.88759 0.59378 0.3988 0.33658
## Proportion of Variance 0.4714 0.2561 0.1232 0.07878 0.03526 0.0159 0.01133
## Cumulative Proportion  0.4714 0.7275 0.8507 0.92945 0.96471 0.9806 0.99193
##                            PC8     PC9    PC10
## Standard deviation     0.18895 0.16194 0.13683
## Proportion of Variance 0.00357 0.00262 0.00187
## Cumulative Proportion  0.99551 0.99813 1.00000
```

```r
#scree plot
plot(prop_varex, xlab = "Principal Component",
       ylab = "Proportion of Variance Explained",
       type = "b")
```

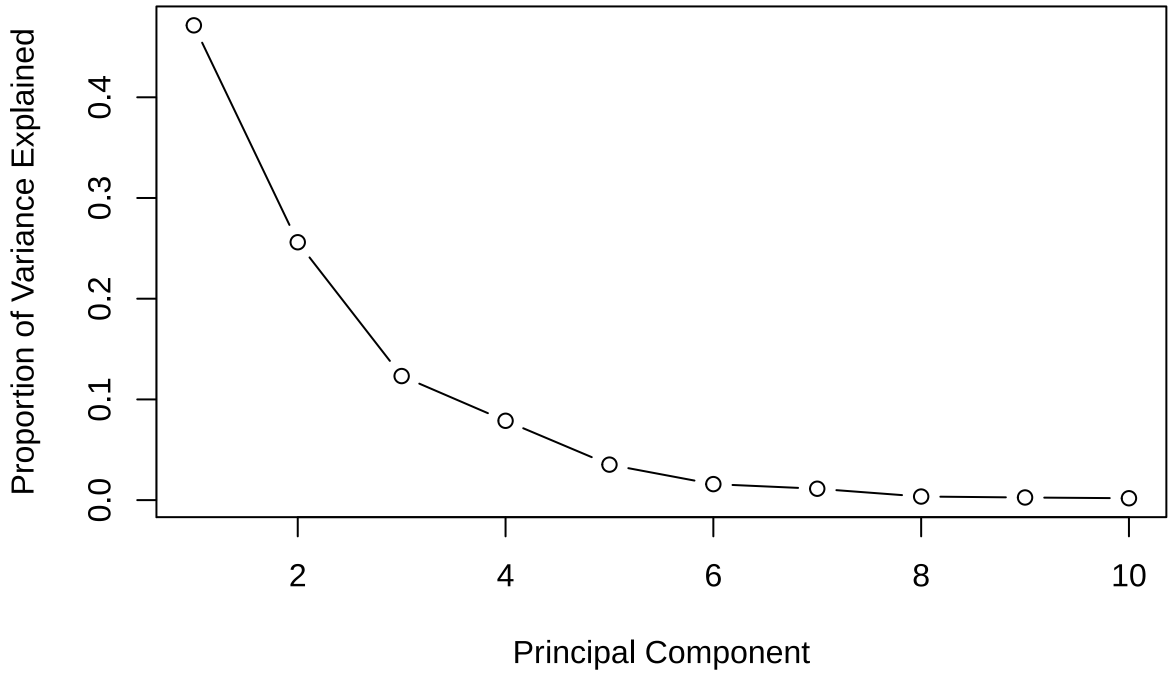


```r
#cumulative scree plot
plot(cumsum(prop_varex), xlab = "Principal Component",
       ylab = "Cumulative Proportion of Variance Explained",
       type = "b")

# alternative plotting with ggplot2
library(ggfortify)
```

```
## Loading required package: ggplot2
```

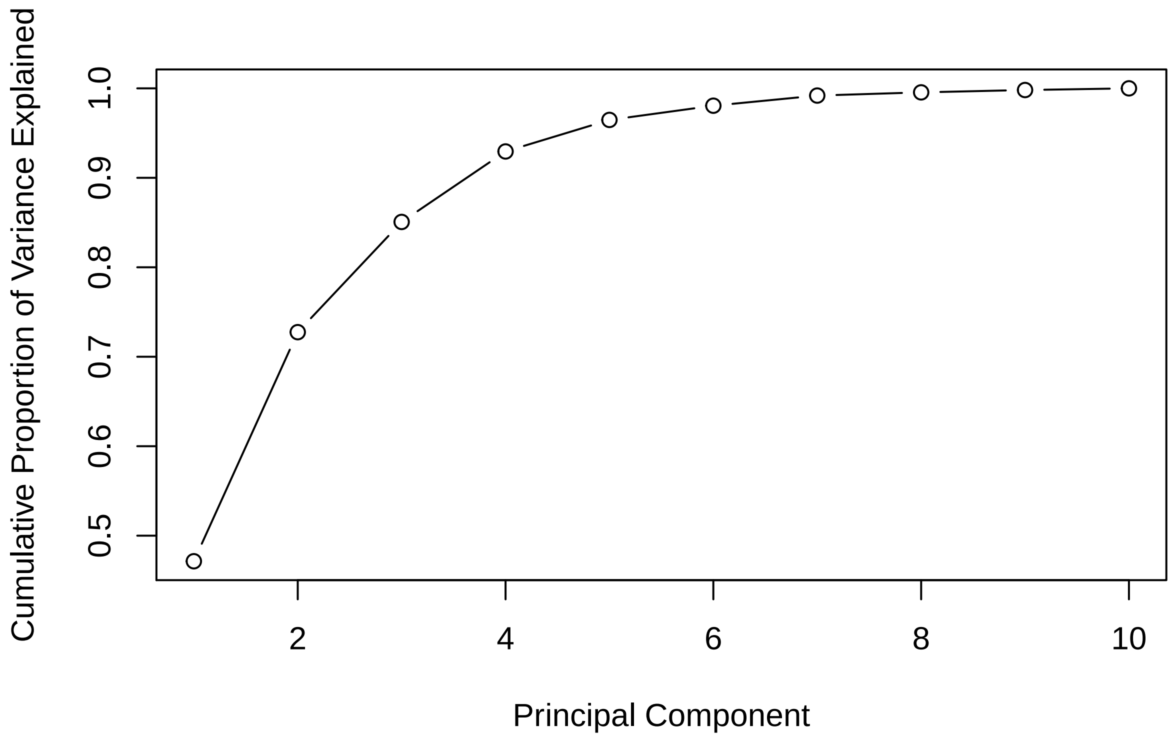


```r
p1<-autoplot(prcomp(sout[,-c(1,12,13)],scale. = T),
             data = sout, colour = 'forage',
             loadings = TRUE, loadings.label = TRUE,
             loadings.label.size = 4,loadings.label.vjust = 1.3,
         loadings.label.hjust = -0.3) + theme_bw() +
  ggtitle('Soutpansberg') + geom_point(aes(shape=forage), size = 4) +
  theme(panel.grid.major = element_blank(),
               panel.grid.minor = element_blank(),
               panel.background = element_blank())
p1
```

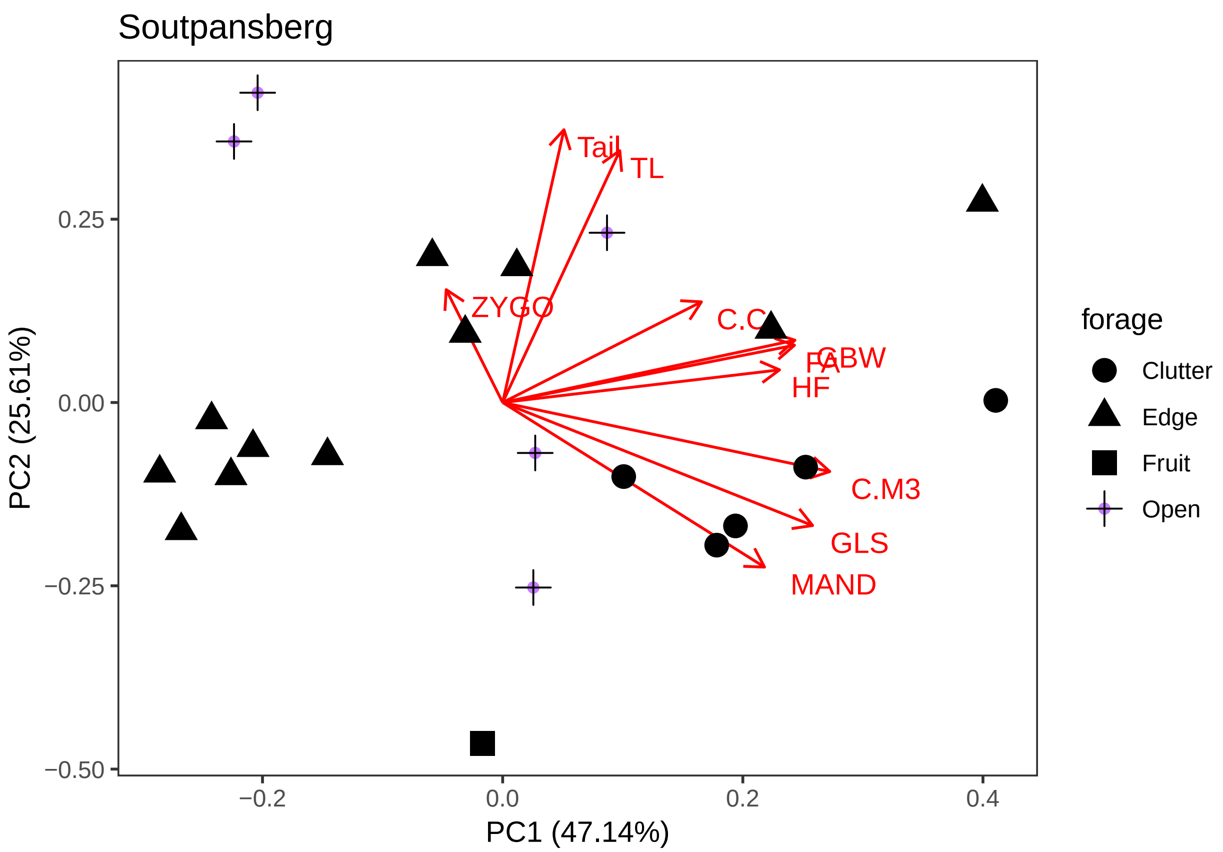


```
# PCA analysis on Nimba bats
```

```
pcaNimba<-prcomp(nimba[,-c(1,12,13)],scale. = T)
# pcaNimba<-prcomp(nimba[,-12])
# plot the PCA
# The parameter scale = 0 ensures that arrows are scaled to represent the loadings.
# biplot(pcaNimba, scale = 0)
# can also plot with the species names
row.names(nimba) <- nimba$Row.Labels
# biplot(pcaNimba<-prcomp(nimba[,-c(1,12,13)],scale. = T))

#compute standard deviation of each principal component
std_dev <- pcaNimba$sdev
#compute variance
pr_var <- std_dev^2
#proportion of variance explained
prop_varex <- pr_var/sum(pr_var)
# remove exponential notation
options(scipen = 999)
# show proportion of variance explained
prop_varex
```

```
##  [1] 0.687272173 0.191528589 0.042415836 0.026620416 0.023478690
##  [6] 0.011037137 0.006181551 0.005224453 0.004130309 0.002110845
```

```
# alternatively just run summary
summary(pcaNimba)
```

```
## Importance of components:
##                           PC1    PC2     PC3     PC4     PC5     PC6
## Standard deviation     2.6216 1.3839 0.65127 0.51595 0.48455 0.33222
## Proportion of Variance 0.6873 0.1915 0.04242 0.02662 0.02348 0.01104
## Cumulative Proportion  0.6873 0.8788 0.92122 0.94784 0.97132 0.98235
##                            PC7     PC8     PC9    PC10
## Standard deviation     0.24863 0.22857 0.20323 0.14529
## Proportion of Variance 0.00618 0.00522 0.00413 0.00211
## Cumulative Proportion  0.98853 0.99376 0.99789 1.00000
```

```
#scree plot
plot(prop_varex, xlab = "Principal Component",
     ylab = "Proportion of Variance Explained",
     type = "b")
```

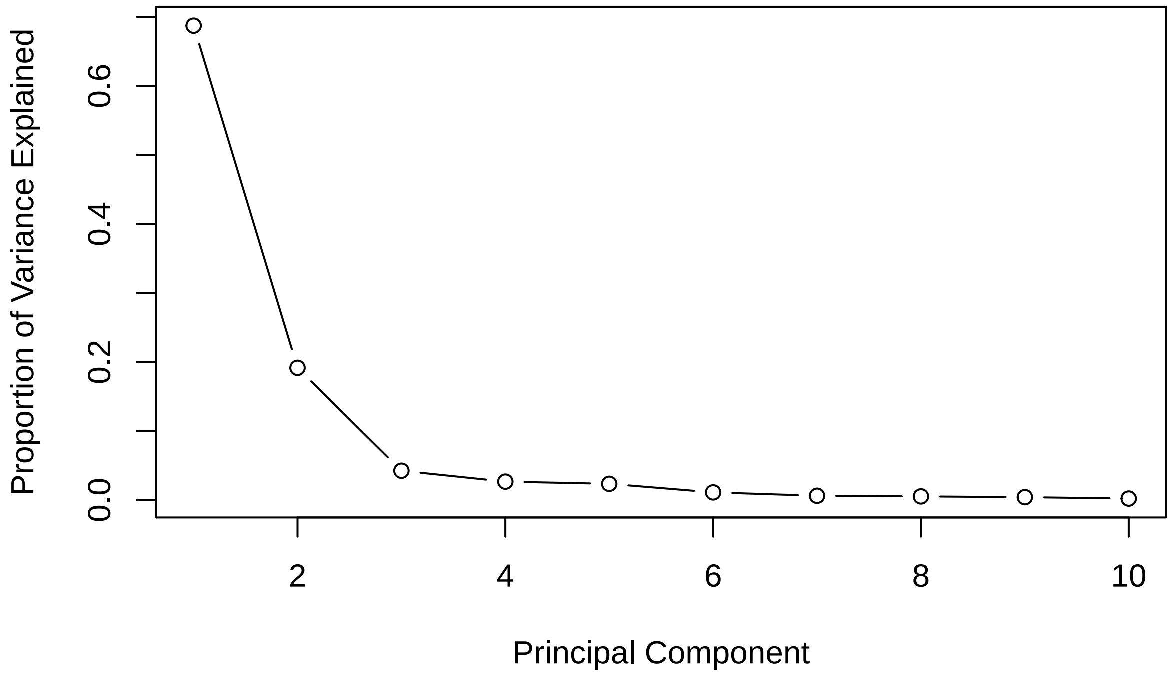


```
#cumulative scree plot
plot(cumsum(prop_varex), xlab = "Principal Component",
     ylab = "Cumulative Proportion of Variance Explained",
     type = "b")
```

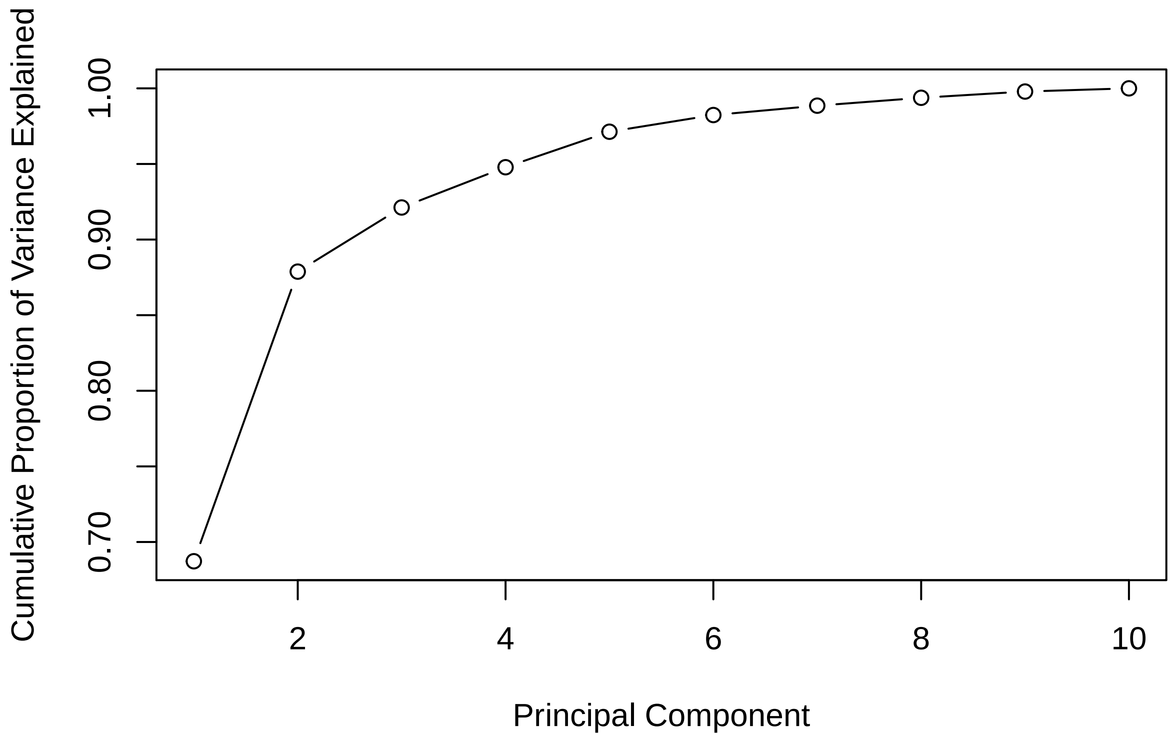


```
p2<-autoplot(prcomp(nimba[,-c(1,12,13)],scale. = T),
             data = nimba, colour = 'forage',
             loadings = TRUE, loadings.label = TRUE,
             loadings.label.size = 4,loadings.label.vjust = 0.8,
         loadings.label.hjust = -0.6) + theme_bw() +
  ggtitle('Nimba')  + geom_point(aes(shape=forage), size = 4) +
  theme(panel.grid.major = element_blank(),
        panel.grid.minor = element_blank(),
        panel.background = element_blank())
p2
```

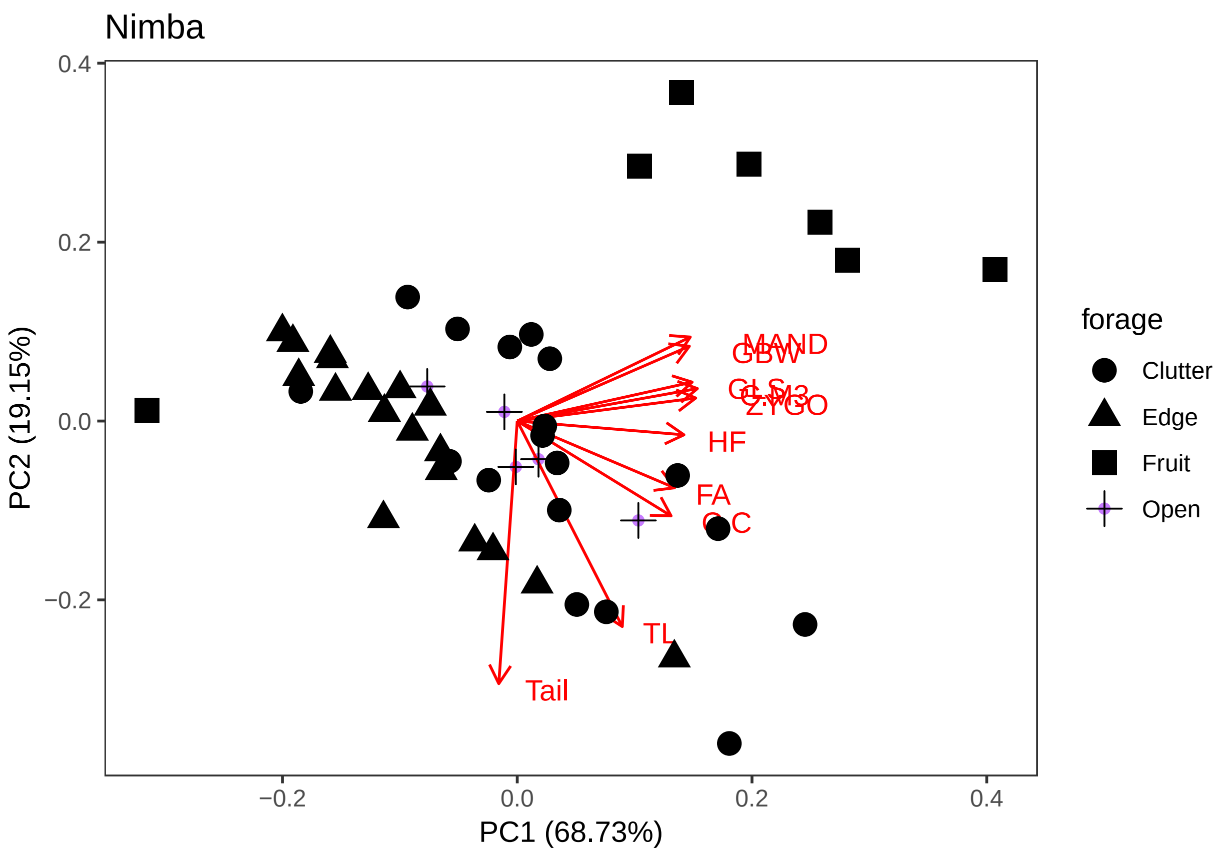


```r
# nearest neighbour - Soutpansberg
```

```r
# coordinates for plot
# pcaSout$x
# PC1 = x axis
# pcaSout$x[,1]
# PC2 = y axis
# pcaSout$x[,2]
# plot them
# plot(pcaSout$x[,1], pcaSout$x[,2])

# spatstat for nearest neighbour distances
library(spatstat)
```

```
## Loading required package: spatstat.data

## Loading required package: nlme

## Loading required package: rpart

##
## spatstat 1.56-1       (nickname: 'Invisible Friend')
## For an introduction to spatstat, type 'beginner'

##
## Note: spatstat version 1.56-1 is out of date by more than 4 months; we recommend upgrading to the la
```

```r
nndistSout<-nndist(pcaSout$x[,1], pcaSout$x[,2]);nndistSout
```

```
##  [1] 0.7885805 1.6537718 0.7237729 0.7885805 0.8098403 0.7237729 0.8400812
##  [8] 0.5375319 1.4430161 2.0330232 0.3439454 0.6369050 0.4520698 0.6166178
## [15] 0.3439454 0.6050155 0.2530534 0.8461350 0.2530534 1.7514394 0.5375319
## [22] 1.3701536
```

```
sd(nndist(pcaSout$x[,1], pcaSout$x[,2]))
```

```
## [1] 0.5002109
```

```
summary(nndist(pcaSout$x[,1], pcaSout$x[,2]))
```

```
##    Min. 1st Qu.  Median    Mean 3rd Qu.    Max.
##  0.2531  0.5375  0.7238  0.8342  0.8446  2.0330
```

```
# test the ppx function here
# This code takes the first two axes and calculates nnds
# it should be compared to the nndistSout object above to see if they match
testppxSout <- ppx(data=data.frame(cbind(pcaSout$x[,1],pcaSout$x[,2])))
(nndistTestppxSout <- nndist(testppxSout))
```

```
##  [1] 0.7885805 1.6537718 0.7237729 0.7885805 0.8098403 0.7237729 0.8400812
##  [8] 0.5375319 1.4430161 2.0330232 0.3439454 0.6369050 0.4520698 0.6166178
## [15] 0.3439454 0.6050155 0.2530534 0.8461350 0.2530534 1.7514394 0.5375319
## [22] 1.3701536
```

```
# do this for all axes rather than the first two
# https://www.rdocumentation.org/packages/spatstat/versions/1.56-1/topics/nndist.ppx
soutAllAxes <- ppx(data=data.frame(pcaSout$x))
nndistSoutAllAxes <- nndist(soutAllAxes)
summary(nndistSoutAllAxes)
```

```
##    Min. 1st Qu.  Median    Mean 3rd Qu.    Max.
##  0.6785  1.2935  1.4552  1.8709  2.3033  4.9326
```

```
# nearest neighbour - Nimba
```

```
# pcaNimba$x
# PC1 = x axis
# pcaNimba$x[,1]
# PC2 = y axis
# pcaNimba$x[,2]
# plot them
# plot(pcaNimba$x[,1], pcaNimba$x[,2])

nndistNimba<-nndist(pcaNimba$x[,1], pcaNimba$x[,2]); nndistNimba
```

```
##  [1] 0.26270359 2.28952809 0.29863436 1.03232160 0.77492032 0.84646045
##  [7] 1.69318615 0.29124866 0.84410170 0.10675914 0.10675914 2.25949341
## [13] 0.34948310 0.16042282 0.16357290 0.58728309 1.01714870 1.26471123
## [19] 0.34301660 0.65287249 0.29863436 0.77586684 0.61604815 0.21452526
## [25] 0.36005322 0.58728309 0.21452526 1.01714870 0.19875941 0.06790763
## [31] 0.38643626 0.34997915 0.16357290 0.17334797 0.06790763 0.50678958
## [37] 1.25129536 0.16042282 0.44274617 0.46296738 0.46296738 0.34301660
## [43] 0.19875941 0.38745416 0.77586684 0.35849093 0.35849093 1.16258618
```

```
sd(nndist(pcaNimba$x[,1], pcaNimba$x[,2]))
```

```
## [1] 0.5119376
```

```r
summary(nndist(pcaNimba$x[,1], pcaNimba$x[,2]))
```

```
##    Min. 1st Qu.  Median    Mean 3rd Qu.    Max. 
## 0.06791 0.21453 0.37324 0.57726 0.77587 2.28953
```

```r
# test the ppx function here
# This code takes the first two axes and calculates nnds
# it should be compared to the nndistSout object above to see if they match
testppxNimba <- ppx(data=data.frame(cbind(pcaNimba$x[,1],pcaNimba$x[,2])))
(nndistTestppxNimba <- nndist(testppxNimba))
```

```
##  [1] 0.26270359 2.28952809 0.29863436 1.03232160 0.77492032 0.84646045
##  [7] 1.69318615 0.29124866 0.84410170 0.10675914 0.10675914 2.25949341
## [13] 0.34948310 0.16042282 0.16357290 0.58728309 1.01714870 1.26471123
## [19] 0.34301660 0.65287249 0.29863436 0.77586684 0.61604815 0.21452526
## [25] 0.36005322 0.58728309 0.21452526 1.01714870 0.19875941 0.06790763
## [31] 0.38643626 0.34997915 0.16357290 0.17334797 0.06790763 0.50678958
## [37] 1.25129536 0.16042282 0.44274617 0.46296738 0.46296738 0.34301660
## [43] 0.19875941 0.38745416 0.77586684 0.35849093 0.35849093 1.16258618
```

```r
# do this for all axes rather than the first two
# https://www.rdocumentation.org/packages/spatstat/versions/1.56-1/topics/nndist.ppx
nimbaAllAxes <- ppx(data=data.frame(pcaNimba$x))
nndistNimbaAllAxes <- nndist(nimbaAllAxes)
summary(nndistNimbaAllAxes)
```

```
##    Min. 1st Qu.  Median    Mean 3rd Qu.    Max. 
##  0.3920  0.6272  0.9990  1.1877  1.3372  2.9369
```

```r
# compare NND for Nimba Vs NND for Soutpansberg
```

```r
wilcox.test(nndistNimbaAllAxes, nndistSoutAllAxes)
```

```
## Warning in wilcox.test.default(nndistNimbaAllAxes, nndistSoutAllAxes):
## cannot compute exact p-value with ties
```

```
## 
##  Wilcoxon rank sum test with continuity correction
## 
## data:  nndistNimbaAllAxes and nndistSoutAllAxes
## W = 278, p-value = 0.001595
## alternative hypothesis: true location shift is not equal to 0
```

```r
# plot the two areas using only PC1 and PC2
```

```r
# plot(pcaNimba$x[,1], pcaNimba$x[,2],col="red",main = "Nimba")
# plot(pcaSout$x[,1], pcaSout$x[,2],main = "Soutpansberg")
```

```r
# convex-hull of morphospace
```

```r
# test on simulated data first
# write the function
cha<-function(x,y){
  chull(x,y)->i
  return(areapl(cbind(x[i],y[i])))
}
library(splancs)
```

```
## Loading required package: sp
```

```
##
## Spatial Point Pattern Analysis Code in S-Plus
##
##  Version 2 - Spatial and Space-Time analysis
```

```r
x<-rnorm(20);rnorm(20)->y;
#Some visualization
# i<-chull(x,y);plot(x,y);polygon(x[i],y[i]);
#The area
# cha(x,y)

# for Nimba
cha(pcaNimba$x[,1],pcaNimba$x[,2])
```

```
## [1] 46.68351
```

```r
i<-chull(pcaNimba$x[,1],pcaNimba$x[,2]);plot(pcaNimba$x[,1],pcaNimba$x[,2]);polygon(pcaNimba$x[,1][i],pc
```

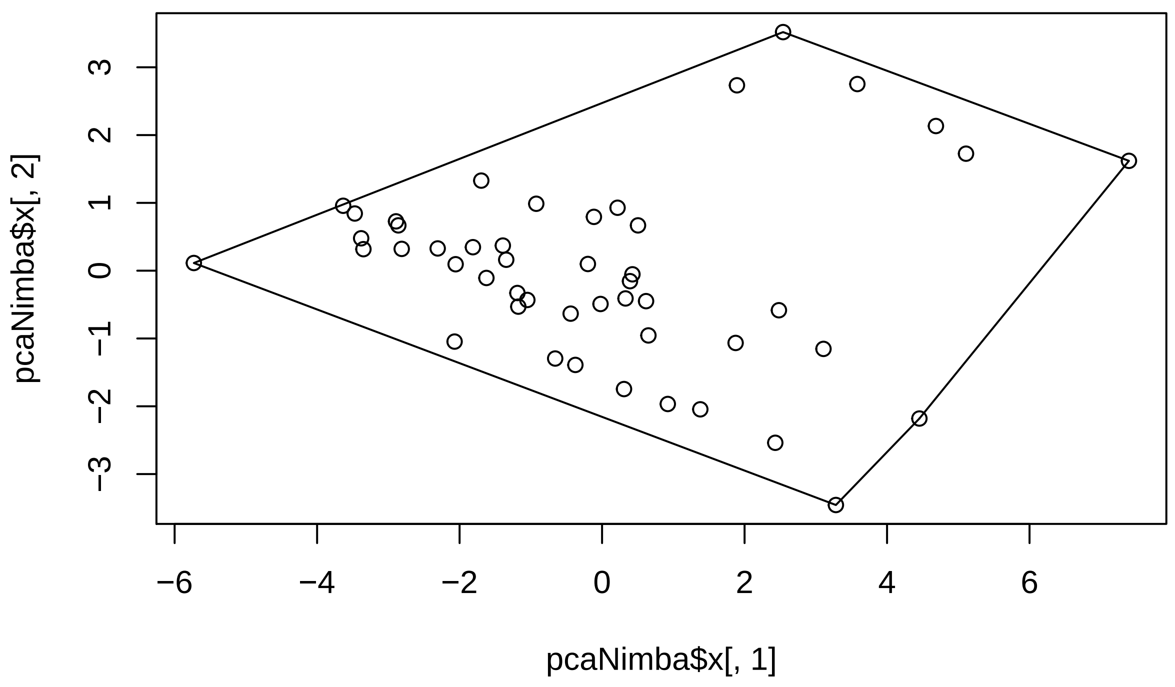


```r
# for Soutpansberg
cha(pcaSout$x[,1],pcaSout$x[,2])
```

```
## [1] 31.23512
```

```r
i<-chull(pcaSout$x[,1],pcaSout$x[,2]);plot(pcaSout$x[,1],pcaSout$x[,2]);polygon(pcaSout$x[,1][i],pcaSou
```

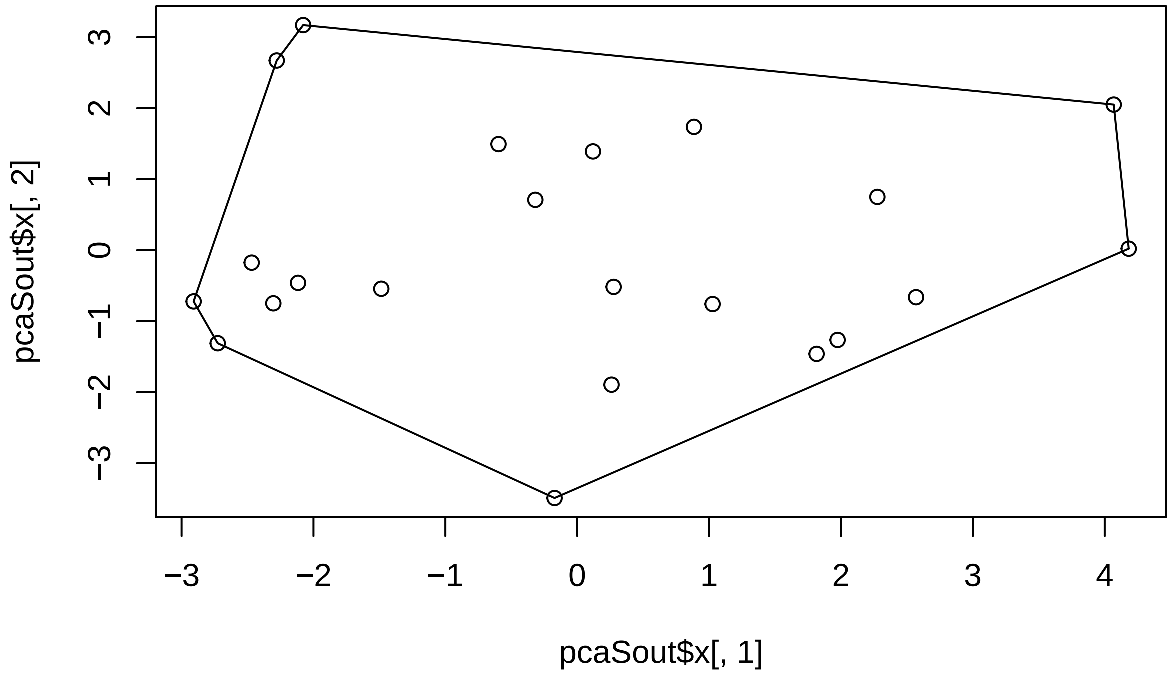


```r
# alternative way to calculate area
```

```r
library(sp)
# test
x1 <- rnorm(100, 0.8, 0.3)
y1 <- rnorm(100, 0.8, 0.3)
hpts <- chull(x = x1, y = y1)
hpts <- c(hpts, hpts[1])
xy.coords <- cbind(x1, y1)
chull.coords <- xy.coords[hpts,]
chull.poly <- Polygon(chull.coords, hole=F)
chull.area <- chull.poly@area

# Nimba
hpts <- chull(x = pcaNimba$x[,1], y = pcaNimba$x[,2])
hpts <- c(hpts, hpts[1])
xy.coords <- cbind(pcaNimba$x[,1], pcaNimba$x[,2])
chull.coordsNimba <- xy.coords[hpts,]
chull.poly <- Polygon(chull.coordsNimba, hole=F)
chull.area <- chull.poly@area
chull.area
```

```
## [1] 46.68351
```

```r
# Soutpansberg
hpts <- chull(x = pcaSout$x[,1], y = pcaSout$x[,2])
hpts <- c(hpts, hpts[1])
```

```
xy.coords <- cbind(pcaSout$x[,1], pcaSout$x[,2])
chull.coordsSout <- xy.coords[hpts,]
chull.poly <- Polygon(chull.coordsSout, hole=F)
chull.area <- chull.poly@area
chull.area
```

```
## [1] 31.23512
```

```
# dispRity analysis of two bat communities
# Guillerme, T. (2018). dispRity: a modular R package for measuring disparity. Methods in Ecology and E
# Analyse bat morphospace again with the residual data
# Residuals come from a regression of each trait ~ body mass
```

```
library(dispRity)
```

```
## Loading required package: ape
```

```
##
## Attaching package: 'ape'
```

```
## The following object is masked from 'package:splancs':
##
##     zoom
```

```
## The following objects are masked from 'package:spatstat':
##
##     edges, rotate
```

```
mydata<-read.csv("residBatData.csv",header = T,sep = ",")
head(mydata)
```

```
##   Mass         FA         TL       Tail         HF        GLS        ZYGO
## 1 12.0 -3.9175610 -10.469611  -4.469339 -1.1854227  0.5406224  -1.3025166
## 2 78.5 -7.4577093 -23.620975 -22.386064  1.3440258  2.1468374 -10.3578276
## 3 19.8 -0.3485859  11.153387  14.489300 -0.6715927 -1.6039569  -1.2930644
## 4  6.4  7.9226620  -5.157917  -6.944773  0.8815711  1.9867306  -0.1741747
## 5  6.6 -2.3037745  11.145237   5.090064  0.8434642 -0.1031304  -0.5598297
## 6  8.9  4.2422053  16.581506  14.790688  1.4052346 -1.2815320  -1.8098631
##           GBW      C.M3        C.C        MAND              Row.Labels
## 1  0.71399826  0.2158539  0.6536821  0.03940742     Chaerephon_pumilus
## 2 -0.86087844  0.1296216 -0.8099869  3.09304991   Epomophorus_wahlbergi
## 3 -0.16919028 -0.8384861  0.4367254 -0.67381948   Eptesicus_hottentotus
## 4  0.58167209  0.3774314 -0.6692721  0.27941647     Hipposideros_caffer
## 5 -0.09610197 -0.6119107 -0.2338095 -0.40451242     Laephotis_botswanae
## 6 -0.19550372 -0.1993443 -0.3909890 -0.32969471 Miniopterus_natalensis
##    forage location fruit
## 1    Open     sout     0
## 2   Fruit     sout     1
## 3    Edge     sout     0
## 4 Clutter     sout     0
## 5    Edge     sout     0
## 6    Edge     sout     0
```

```
# remove fruit bats if needed
# mydata<-mydata[!(mydata$fruit==1),]
measurements <- mydata[,2:11]
ordination <- prcomp(measurements)
```

```r
## The bat-space
bat_space <- ordination$x

## Adding the elements names to the bat_space (the individuals IDs)
rownames(bat_space) <- 1:nrow(bat_space)
length(bat_space)
```

```
## [1] 700
```

```r
## Creating the table that contain the elements and their attributes
bat_subsets <- custom.subsets(bat_space, group = list(
  "sout" = which(mydata$location == "sout"),
  "nimba" = which(mydata$location == "nimba")))

## Visualising the dispRity object content
bat_subsets
```

```
##  ---- dispRity object ----
## 2 customised subsets for 70 elements:
##     sout, nimba.
```

```r
## Bootstrapping the data
(bat_bootstrapped <- boot.matrix(bat_subsets, bootstraps = 500 ))
```

```
##  ---- dispRity object ----
## 2 customised subsets for 70 elements with 10 dimensions:
##     sout, nimba.
## Data was bootstrapped 500 times (method:"full").
```

```r
# can include more bootstraps and rareify the data so that we're comparing equal sample sizes
# rareify the data,  the number of elements is set to the value(s) for each bootstrap.
min_sample <- length(which(mydata$location == "sout") )
(bat_bootstrapped <- boot.matrix(bat_subsets, bootstraps = 500, rarefaction =  min_sample))
```

```
##  ---- dispRity object ----
## 2 customised subsets for 70 elements with 10 dimensions:
##     sout, nimba.
## Data was bootstrapped 500 times (method:"full") and rarefied to 22 elements.
```

```r
## calculates the Euclidean distance between each row and the centroid of the matrix i.e. bat-space
(bat_disparity <- dispRity(bat_bootstrapped, metric = c(centroids)))
```

```
##  ---- dispRity object ----
## 2 customised subsets for 70 elements with 10 dimensions:
##     sout, nimba.
## Data was bootstrapped 500 times (method:"full") and rarefied to 22 elements.
## Disparity was calculated as: c(centroids).
```

```r
## Displaying the summary of the calculated disparity
summary(bat_disparity)
```

```
##   subsets  n obs.median bs.median  2.5%   25%   75% 97.5%
## 1    sout 22      18.25     17.59 7.657 13.05 24.33 41.53
## 2   nimba 48      21.09     19.97 6.710 12.84 27.39 45.73
## 3   nimba 22         NA     19.40 6.343 12.68 27.36 46.94
```

```r
## Graphical options
par(bty = "n")
```

```r
## Plotting the disparity in the bat_space of the rarefied data
plot(bat_disparity, rarefaction = min_sample,ylim = c(0,100))
```

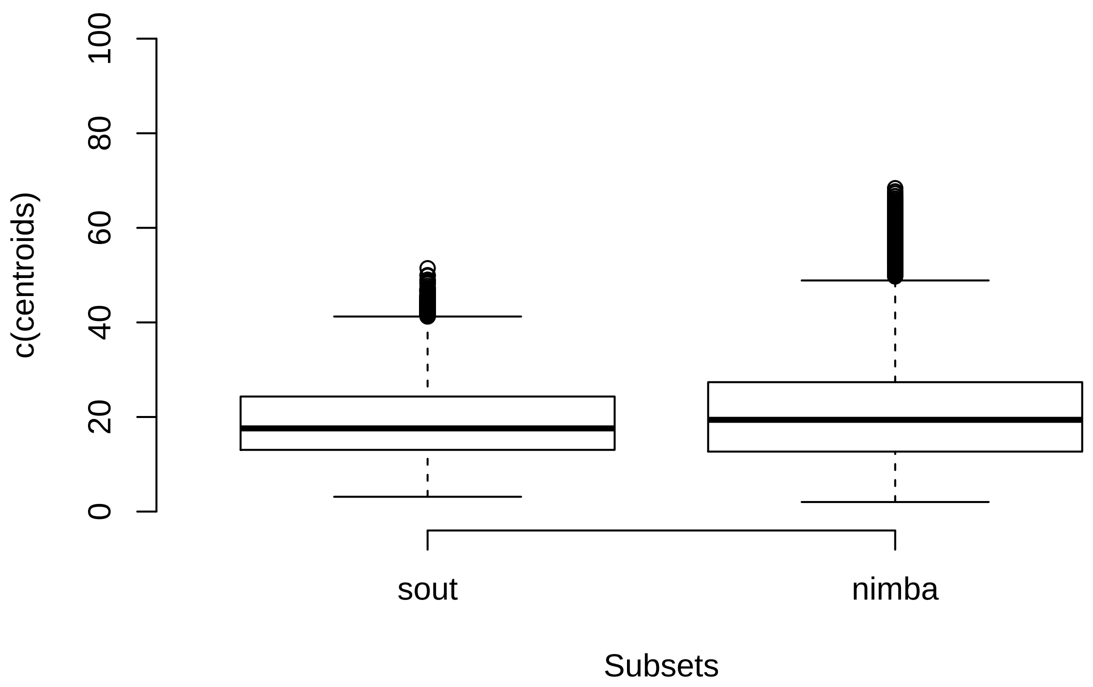


```r
## Running a significance test
sig.test.rare<-suppressWarnings(test.dispRity(bat_disparity, test = wilcox.test, rarefaction = min_sampl
```

```
## [[1]]
##               statistic: W    2.5% 25% 75% 97.5%
## sout : nimba      227.076 132.475 192 262   320
##
## [[2]]
##                 p.value       2.5%       25%       75%     97.5%
## sout : nimba 0.4415005 0.01046679 0.1768986 0.7157989 0.9817341
```

```r
# Compare to the non bootstrapped and non rarefied sample
```

```r
(bat_disparityFull <- dispRity(bat_subsets, metric = c(centroids)))
```

```
##  ---- dispRity object ----
## 2 customised subsets for 70 elements with 10 dimensions:
##     sout, nimba.
## Disparity was calculated as: c(centroids).
```

```r
## Displaying the summary of the calculated disparity
summary(bat_disparityFull)
```

```
##   subsets  n obs.median  2.5%   25%   75% 97.5%
## 1    sout 22      18.25 10.25 14.39 24.00 40.52
```

```
## 2   nimba 48      21.09   6.93 13.68 26.42 44.96
```

```r
## Graphical options
par(bty = "n")

## Plotting the disparity in the bat_space of the rarefied data
plot(bat_disparityFull, rarefaction = min_sample,ylim = c(0,100))
```

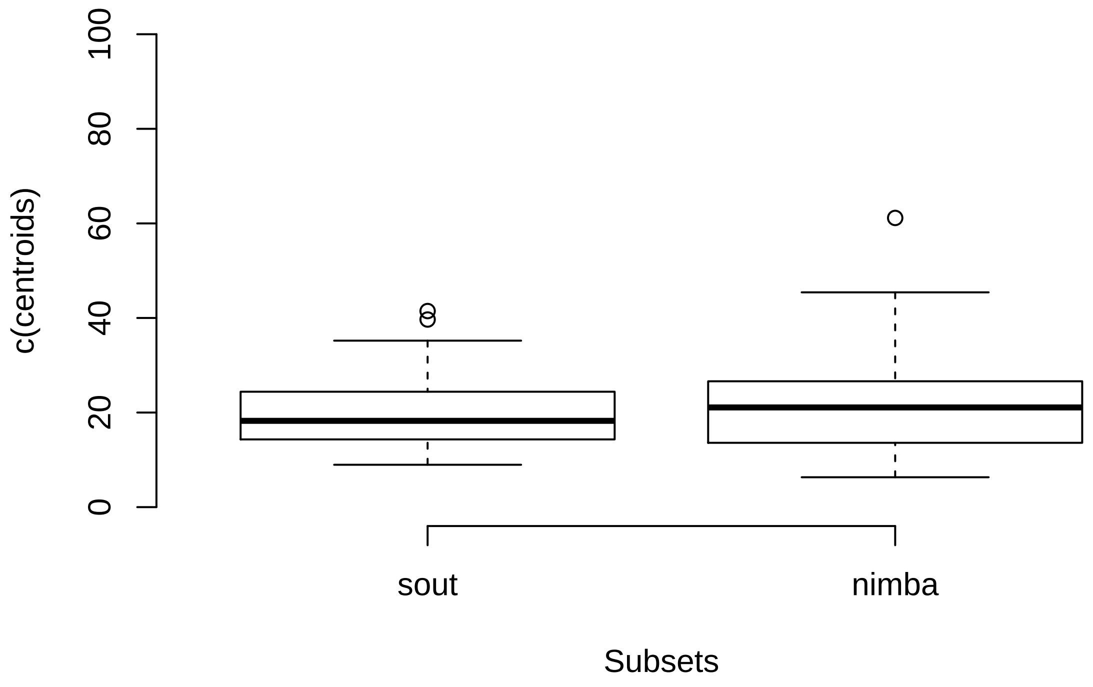


```r
## Running a significance test
sig.test<-suppressWarnings(test.dispRity(bat_disparityFull, test = wilcox.test) ); sig.test
```

```
## [[1]]
##                statistic: W
## sout : nimba          500
##
## [[2]]
##                  p.value
## sout : nimba 0.7298833
```
