# Supplementary material for: Morphology and stable isotope analysis demonstrate different structuring of bat communities in rainforest and savannah habitats
Source: R Soc Open Sci. 2018 Dec 19;5(12):180849. doi: 10.1098/rsos.180849 (PMC6304110; doi:10.1098/rsos.180849)
Supplement: Supplementary Tables [file rsos180849supp2.docx]

Table S1. The bat families, species and sample sizes used for the species packing (morphometric) and niche breadth (stable isotope) analyses in rainforest (Nimba) or savanna (RSA).

| Family and species | Number of specimens used in each analysis | | | |
| --- | --- | --- | --- | --- |
|  | Species packing (Nimba) | Niche breadth (Nimba | Species packing (RSA) | Niche breadth (RSA) |
| **Emballonuridae** |  |  |  |  |
| *Taphozous mauritianus* | 0 | 0 | 1 | 1 |
| **Hipposideridae** |  |  |  |  |
| *Doryrhina cyclops* | 1 | 1 | 0 | 0 |
| *Hipposideros beatus* | 4 | 3 | 0 | 0 |
| *Hipposideros caffer* | 0 | 0 | 2 | 2 |
| *Hipposideros lamottei* | 5 | 0 | 0 | 0 |
| *Hipposideros marisae* | 1 | 1 | 0 | 0 |
| *Hipposideros ruber (lineage C1)* | 4 | 2 | 0 | 0 |
| *Hipposideros ruber (lineage E1)* | 5 | 1 | 0 | 0 |
| *Macronycteris gigas* | 1 | 1 | 0 | 0 |
| **Miniopteridae** |  |  |  |  |
| *Miniopterus inflatus* | 3 | 3 | 0 | 0 |
| *Miniopterus natalensis* | 0 | 0 | 4 | 2 |
| *Miniopterus villiersi* | 4 | 3 | 0 | 0 |
| **Molossidae** |  |  |  |  |
| *Chaerephon pumilus* | 2 | 2 | 1 | 1 |
| *Mops condylurus* | 2 | 2 | 1 | 0 |
| *Mops leonis* | 2 | 2 | 0 | 0 |
| *Mops midas* | 0 | 0 | 4 | 0 |
| *Mops spurrelli* | 2 | 2 | 0 | 0 |
| *Mops thersites* | 2 | 2 | 0 | 0 |
| *Tadarida ventralis* | 0 | 0 | 1 | 0 |
| **Nycteridae** |  |  |  |  |
| *Nycteris arge* | 2 | 2 | 0 | 0 |
| *Nycteris grandis* | 1 | 1 | 0 | 0 |
| *Nycteris hispida* | 1 | 1 | 0 | 0 |
| *Nycteris intermedia* | 1 | 0 | 0 | 0 |
| *Nycteris macrotis* | 1 | 1 | 0 | 0 |
| *Nycteris major* | 1 | 0 | 0 | 0 |
| *Nycteris thebaica* | 0 | 0 | 0 | 1 |
| **Pteropodidae** |  |  |  |  |
| *Epomophorus wahlbergi* | 0 | 0 | 2 | 2 |
| *Epomops buettikoferi* | 1 | 1 | 0 | 0 |
| *Hypsignathus monstrosus* | 1 | 1 | 0 | 0 |
| *Megaloglossus azagny* | 1 | 1 | 0 | 0 |
| *Micropteropus pusillus* | 2 | 2 | 0 | 0 |
| *Myonycteris angolensis* | 3 | 3 | 0 | 0 |
| *Myonycteris leptodon* | 2 | 2 | 0 | 0 |
| *Nanonycteris veldkampii* | 1 | 1 | 0 | 0 |
| **Rhinolophidae** |  |  |  |  |
| *Rhinolophus clivosus* | 0 | 0 | 5 | 3 |
| *Rhinolophus darling* | 0 | 0 | 3 | 2 |
| *Rhinolophus guineensis* | 1 | 1 | 0 | 0 |
| *Rhinolophus hillorum* | 3 | 3 | 0 | 0 |
| *Rhinolophus landeri* | 2 | 0 | 0 | 0 |
| *Rhinolophus simulator* | 1 | 0 | 1 | 1 |
| *Rhinolophus smithersi* | 0 | 0 | 2 | 2 |
| **Vespertilionidae** |  |  |  |  |
| *Eptesicus hottentotus* | 0 | 0 | 3 | 1 |
| *Glauconycteris poensis* | 5 | 3 | 0 | 0 |
| *Glauconycteris sp.* | 1 | 1 | 0 | 0 |
| *Hypsugo bellieri* | 3 | 0 | 0 | 0 |
| *Hypsugo sp.* | 1 | 1 | 0 | 0 |
| *Kerivoula phalaena* | 2 | 2 | 0 | 0 |
| *Laephotis botswanae* | 0 | 0 | 1 | 1 |
| *Mimetillus moloneyi* | 2 | 2 | 0 | 0 |
| *Myotis bocagii* | 1 | 1 | 0 | 0 |
| *Myotis tricolor* | 0 | 0 | 1 | 2 |
| *Myotis welwitschii* | 0 | 0 | 1 | 1 |
| *Neoromicia brunnea* | 7 | 3 | 0 | 0 |
| *Neoromicia capensis* | 0 | 0 | 3 | 0 |
| *Neoromicia guineensis* | 1 | 0 | 0 | 0 |
| *Neoromicia Isabella* | 1 | 1 | 0 | 0 |
| *Neoromicia nana* | 4 | 3 | 1 | 0 |
| *Neoromicia roseveari* | 4 | 2 | 0 | 0 |
| *Neoromicia somalica* | 1 | 1 | 0 | 0 |
| *Neoromicia tenuipinnis* | 2 | 2 | 0 | 0 |
| *Neoromicia zuluensis* | 0 | 0 | 3 | 3 |
| *Nycticeinops schlieffeni* | 0 | 0 | 1 | 0 |
| *Pipistrellus hesperidus* | 0 | 0 | 7 | 3 |
| *Pipistrellus nanulus* | 6 | 3 | 0 | 0 |
| *Pipistrellus rusticus* | 0 | 0 | 3 | 0 |
| *Pipistrellus sp.* | 1 | 0 | 0 | 0 |
| *Scotophilus nux* | 2 | 2 | 0 | 0 |

Table S2. Layman metrics and standard ellipses for bat families in Nimba (rainforest) and Soutpansberg (savanna) mountains.

| Functional group | Region | *δ*^15^N range (NR) | *δ*^13^C range (CR) | Distance to centroid (CD) | Mean nearest neighbour distance (MNND) | SD of nearest neighbour distance (SDNND) | Convex hull (TA) | Area of ellipse (SEA) | Corrected area of ellipse (SEA_C_) | Sample size (# species) |
| --- | --- | --- | --- | --- | --- | --- | --- | --- | --- | --- |
| Open air | Nimba | 3.47 | 6.47 | 2.16 | 1.00 | 0.78 | 16.51 | 7.80 | 8.66 | 11 (5) |
|  | Soutpansberg | 0.83 | 2.49 | 1.31 | 2.63 | - | - | - | - | 2 (2) |
| Edge | Nimba | 5.82 | 12.50 | 1.97 | 0.69 | 0.75 | 37.99 | 8.32 | 8.59 | 33 (16) |
|  | Soutpansberg | 4.82 | 4.67 | 2.03 | 0.75 | 0.47 | 12.51 | 6.37 | 6.95 | 13 (7) |
| Clutter | Nimba | 4.38 | 13.54 | 1.77 | 1.05 | 2.25 | 27.36 | 8.05 | 8.55 | 18 (11) |
|  | Soutpansberg | 4.93 | 3.58 | 1.47 | 0.85 | 0.46 | 9.79 | 4.67 | 5.13 | 13 (8) |
| Fruitbat | Nimba | 4.68 | 3.29 | 1.42 | 0.69 | 0.32 | 8.07 | 3.70 | 4.01 | 14 (7) |
|  | Soutpansberg | 1.12 | 1.40 | 0.80 | 1.59 | - | - | - | - | 2 (1) |
